# Transmission of canine transmissible venereal tumour between two dogs in the United Kingdom

**DOI:** 10.1111/jsap.13607

**Published:** 2023-03-29

**Authors:** AM Hayes, L Schiavo, F Constantino-Casas, I Desmas, J Dobson, A Draper, J Elliot, M-A Genain, J Wang, EP Murchison

**Affiliations:** Dept Veterinary Medicine, University of Cambridge, Madingley Rd. Cambridge, CB3 0ES; Dept Veterinary Medicine, University of Cambridge, Madingley Rd. Cambridge, CB3 0ES; Dept Veterinary Medicine, University of Cambridge, Madingley Rd. Cambridge, CB3 0ES; Manor Farm Business Park, Higham Gobion, Hitchin, SG53HR, UK; Dept Veterinary Medicine, University of Cambridge, Madingley Rd. Cambridge, CB3 0ES; Dept Veterinary Medicine, University of Cambridge, Madingley Rd. Cambridge, CB3 0ES; Dept Veterinary Medicine, University of Cambridge, Madingley Rd. Cambridge, CB3 0ES; No 1 Bramston Way, Southfields, Laindon, Essex, SS15 6TP, UK; Dept Veterinary Medicine, University of Cambridge, Madingley Rd. Cambridge, CB3 0ES; Dept Veterinary Medicine, University of Cambridge, Madingley Rd. Cambridge, CB3 0ES; Dept Veterinary Medicine, University of Cambridge, Madingley Rd. Cambridge, CB3 0ES

## Abstract

Canine transmissible venereal tumour (CTVT) is a contagious cancer spread by transfer of living cancer cells. Occasional cases are observed in the UK in dogs imported from endemic regions. Here we report a case of imported CTVT that was transmitted to a second dog within the UK. Transmission of genital CTVT occurred despite neutered status of the second dog. The aggressive course of disease in both cases, which included metastasis, resistance to therapeutic interventions, and ultimate euthanasia of both dogs, is described. The diagnosis of CTVT was made using a combination of cytology, histology, immunohistochemistry, and PCR to detect the *LINE-MYC* rearrangement. Practitioners unfamiliar with CTVT are reminded of this disease of concern, particularly when imported dogs are placed in multi-dog households, irrespective of neuter status.

## Introduction

Canine transmissible venereal tumour (CTVT) is a contagious cancer that spreads between dogs by the physical transfer of cancer cells usually during mating. CTVT usually manifests as localised genital tumours, and metastasis is observed rarely (Strakova & Murchison 2014). Tumours usually resolve with a course of vincristine chemotherapy (Ganguly *et al*. 2016; Scarpelli *et al*. 2010; Woods 2019). The disease originated in a single “founder dog” (Baez-Ortega *et al*. 2019) and has since spread globally (Strakova & Murchison 2014). Once widespread in the United Kingdom (UK), the disease declined due to the disappearance of free-roaming dogs (Strakova & Murchison 2014). However, CTVT remains common in many parts of the world, including some European countries. Cases in the UK are occasionally observed in dogs imported from endemic areas (Gibson *et al*. 2021; Jarvis 2023; Loeb & Mack 2023; Strakova & Murchison 2014).

### Case Report

**Figure 1:**
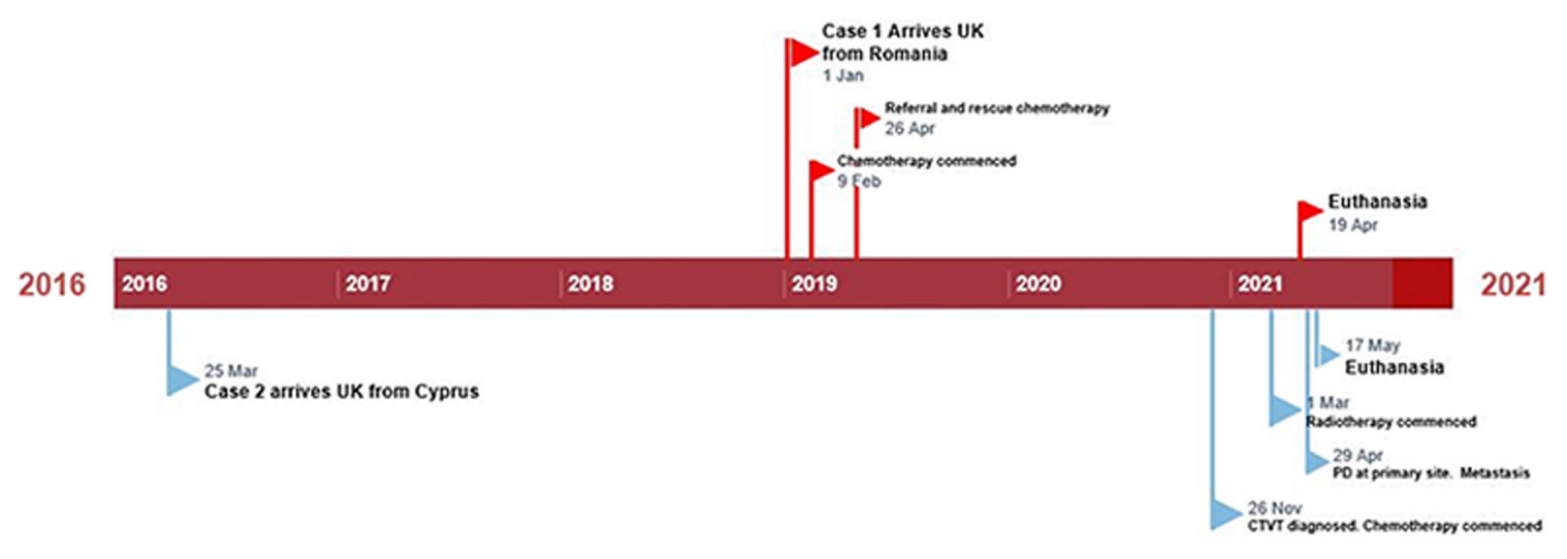
Timeline: Red flags, Case 1; blue flags, Case 2. Rescue chemotherapy pertains to commencement of second line drug treatment with doxorubicin. PD indicates progressive disease.

Two dogs were placed in a single household in the UK between 2016 and 2019 as part of a rehoming initiative (Figure 1).

#### Case 1

**Figure 2:**
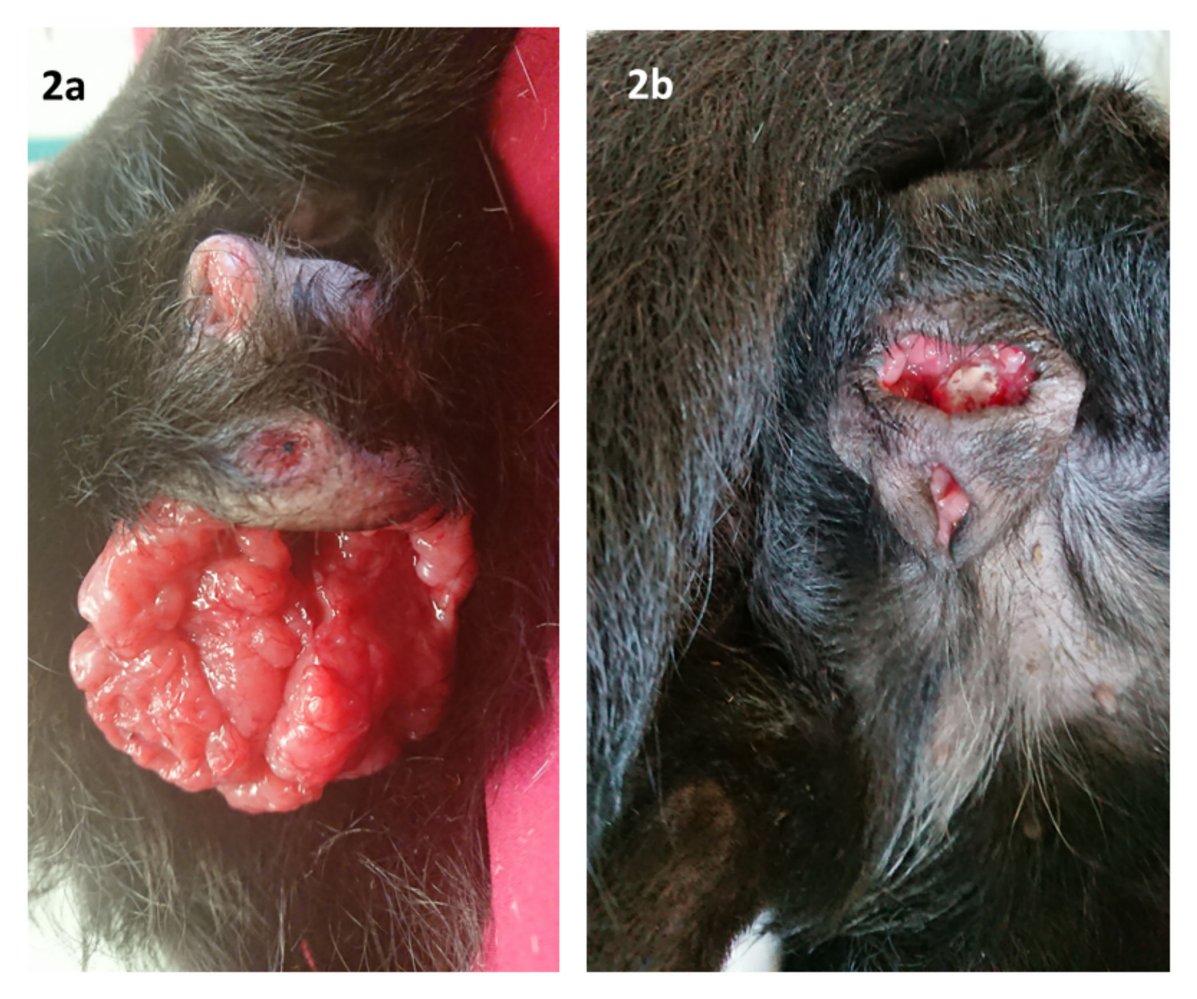
a) Case 1, photograph indicating condition upon arrival to the UK January 2019. Large, irregular mass emerging from the vulval opening. Two additional irregular and lobulated masses are noted dorsally within the perineal skin. b) April 2019, photograph documenting the partial response to vincristine. The erythematous, lobulated mass is noted deep to the open skin edges, the vulval opening is now visible ventrally but a significant skin defect remains in the perineum.

In January 2019, a female entire cross breed bitch aged approximately 5 years and weighing 6.8kg was imported to the UK from Romania (Figure 1). Veterinary examination on the following day documented a large firm irregular lobulated and erythematous vulval mass which obscured the vaginal opening. Two similar masses were noted in haired skin of the perineum (Figure 2a). The attending veterinarian was informed that vincristine and doxorubicin chemotherapy for CTVT had been unsuccessful in Romania.

Following histological confirmation, ten weekly doses of vincristine sulphate (0.6-0.7mg/m^2^ IV. Vincristine 1mg/ml, Hospira UK Ltd, Maidenhead, UK) were administered between January and April 2019. A marked reduction in size of all masses was observed, but residual disease remained (Figure 2b) without further reduction following the latter three treatments.

At referral in April 2019, cytology of a 3cm x 4.8cm mass remaining at the vulval opening confirmed CTVT. This diagnosis was supported by immunohistochemistry (tumour was positive for vimentin, negative for CD3, CD79a and *LINE-MYC* PCR amplification (Figures 5a & 5b)). The mass was associated with a full thickness skin deficit above the vulval opening which communicated with the vagina. CT images of the thorax and the abdomen were obtained. A single, well-defined, soft tissue attenuating, 4.8 mm nodule with an ill-defined surrounding halo was found in the accessory lung lobe. A faint patch of rounded alveolar pattern and a more defined small, rounded, nodular lesion with faintly ill-defined borders was seen in the left and right caudal lung lobes. Although no samples were obtained from lung lesions, metastases at various stages of development were considered the most likely differential.

Medical treatment was attempted between May and July 2019; six doses of doxorubicin (1mg/kg IV. Doxorubicin 2mg/ml, Pfizer Ltd, BVBA, Belgium) were administered at 14-day intervals. Following an initial partial response, local progression occurred. At the owners’ request, two further treatments were given in December 2019 and in July 2020, but these single doses of vincristine and doxorubicin respectively produced no clinical response. Progression of the primary tumour, deteriorating respiratory signs, anorexia and vomiting led to euthanasia in April 2021 on humane grounds.

#### Case 2

In April 2016 an 8-month-old male neutered cross breed dog weighing 8kg was imported to the UK from Cyprus. Although the penis was not specifically examined at the time of arrival into the UK, the dog remained well, showing no signs of CTVT until October 2020, twenty-two months after Case 1 entered the household (Figure 1).

**Figure 3:**
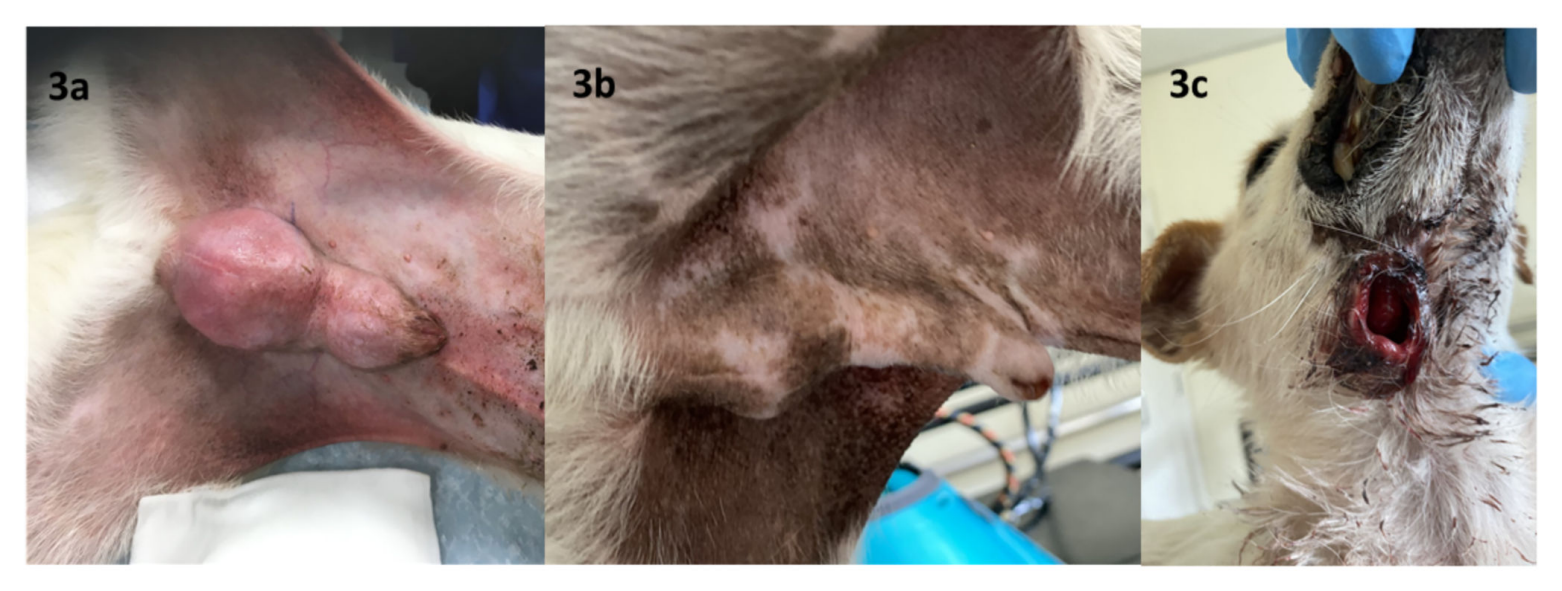
Case 2. Photographs illustrating genital lesion at (a) commencement of radiotherapy and (b) 8 weeks post radiotherapy. Swelling can be seen at the level of the bulbus glandis, at the base of the penis. Note the hair loss and changes in skin pigmentation in the radiation field, and serous blood-stained leakage of fluid at the tip of the prepuce in (b). (c) Cavitating mass within the right ventral neck. Crusting exudate and full thickness skin loss. The pink mass lesion can be seen at the depth of the skin defect within the subdermal tissues.

The dog was presented to a local veterinarian with a marked swelling in the caudal penis and blood staining at the prepuce. The penis could not be exteriorised from the prepuce due to pain and swelling. Histology confirmed CTVT. An elevated serum ALKP 1595 IU/L (reference interval 23-212) remained throughout treatment. A solitary, small liver nodule was noted on abdominal ultrasound, without further investigation. Additional staging was declined by the owner. Five doses of vincristine (0.7mg/m^2^ IV) were administered on a weekly basis during November and December 2020. Despite a modest reduction in size, a bulky mass measuring 4.9cm x 3cm remained. Two doses of doxorubicin (1mg/kg IV) given at three-week intervals produced no measurable response. Referral for radiotherapy was sought and a total of 20Gy in five daily fractions was delivered to the penile lesion during March 2021. A partial response was seen (Figures 3a and 3b). However, a 2cm diameter, ulcerated cutaneous lesion developed in the right ventral neck one month later (Figure 3c).

**Figure 4:**
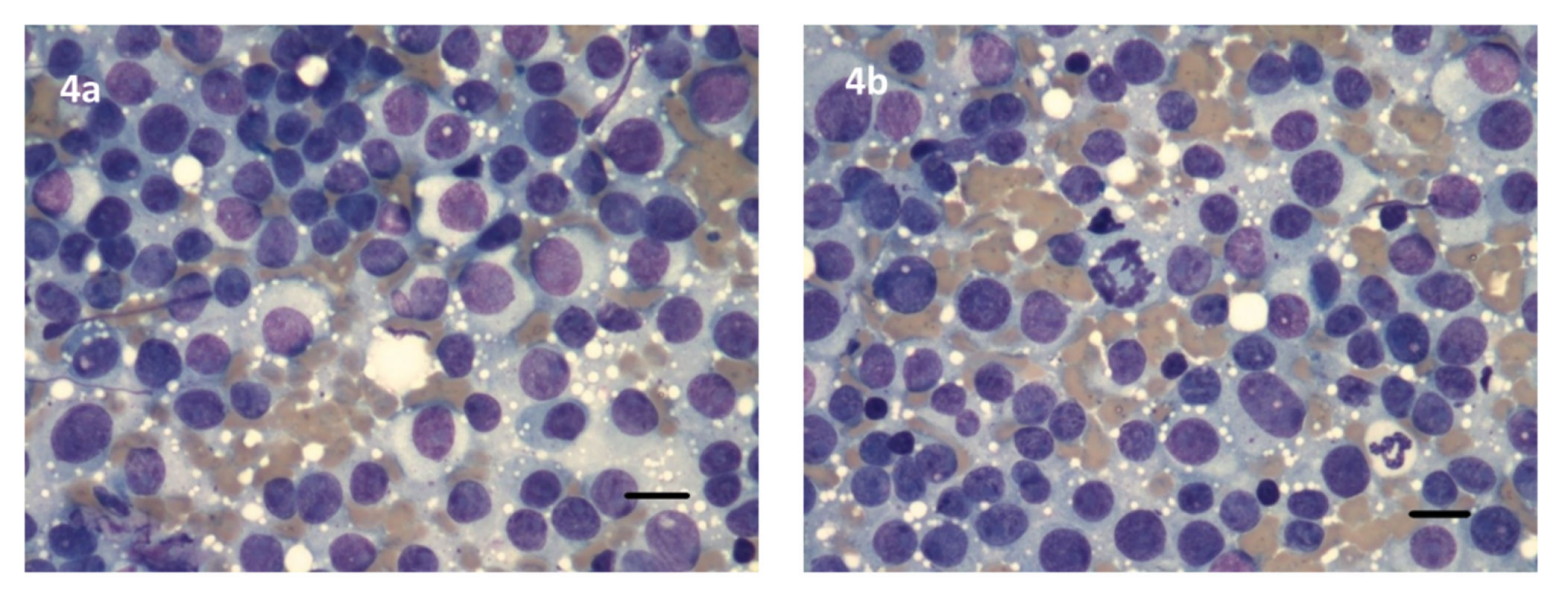
Cytology, Liver mass. Case 2. (a) Monolayer of large (2-3x neutrophil diameter) round cells, often with a naked nuclear appearance, were seen, with a large oval nucleus and abundant light blue cytoplasm often containing discrete vacuoles. Small lymphocytes were present in low numbers. (b). Mitotic figures were frequently seen in some areas. Cytology was consistent with CTVT. Wright Giemsa. Scale bar, 50 um.

**Figure 5:**
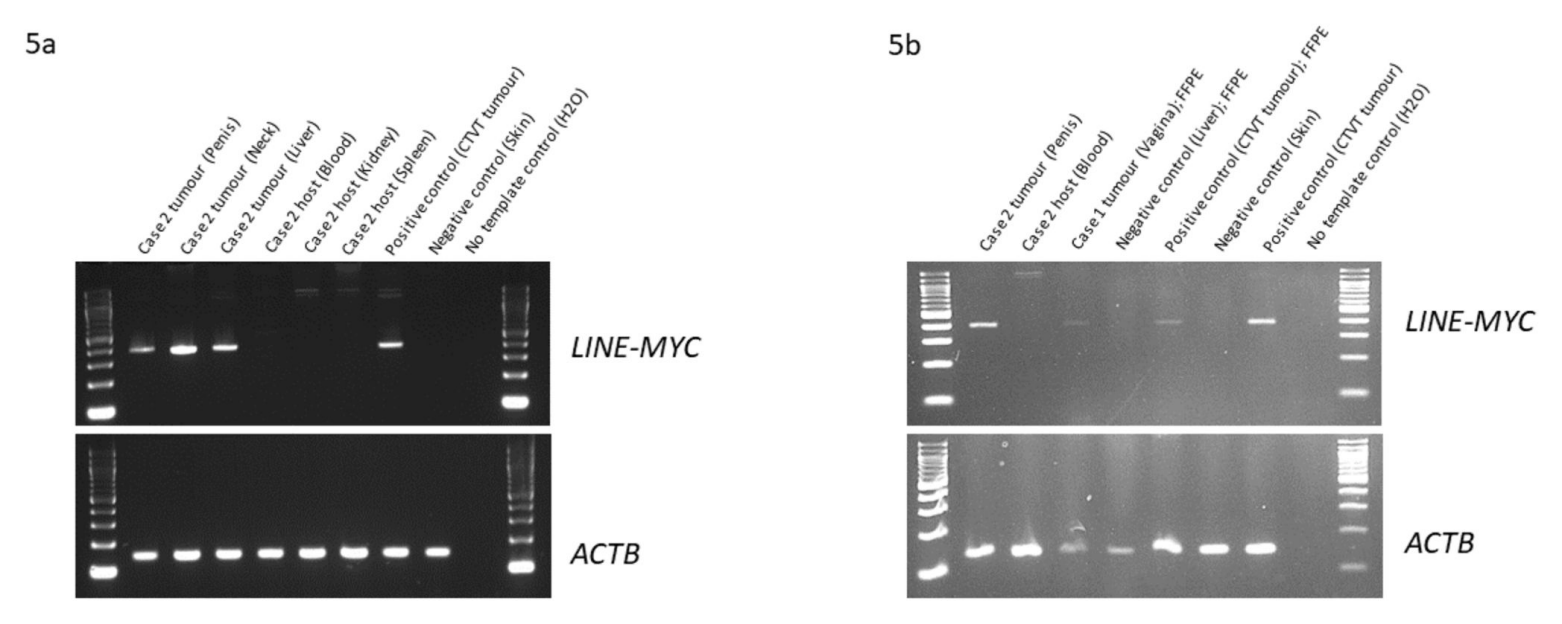
CTVT diagnostic *LINE-MYC* PCR of samples from Cases 1 and 2. M: 100bp DNA ladder (New England BioLabs). (Murgia *et al*. 2006).

Following a second referral, the dog was dull but responsive, weighed 7.4kg with a body condition score (BCS) 3/9. Both the penile and neck masses were evident (Figures 3b & 3c). Three view thoracic radiographs showed no evidence of metastasis. An abdominal ultrasound revealed multiple, ill-defined, rounded to multilobulated, hypoechoic masses within all liver lobes, between 1.56 and 3.46cm diameter. Serum alkaline phosphatase remained elevated at 1700iu/L (26-107). There was a lymphocytopenia 0.7 x 10^9^/L (reference interval 1-4.7) and an erythrocytosis (RBC 9.16 X 10^12^/L (5.5-8.5), Hb 22.4 (12-18) HCT 60.9% (37-55)) which was unexplained. Needle aspirate cytology of the liver mass was supportive of CTVT (Figures 4a & 4b). *LINE-MYC* PCR confirmed CTVT in the liver nodules and skin lesion (Figures 5a & 5b).

**Figure 6:**
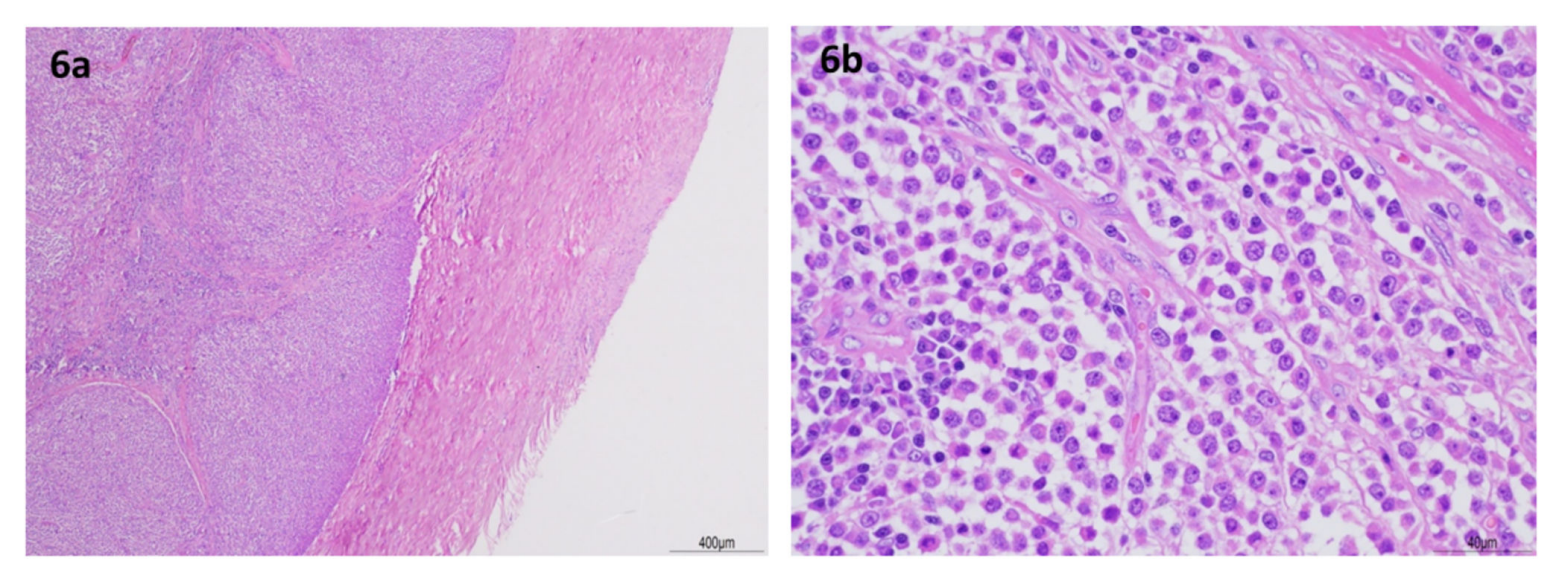
Figure 6a. Histopathology was consistent with CTVT. Case 2, penis. Neoplastic cells are arranged in lobules supported by a fibrovascular collagenous stroma. (H&E) Figure 6b. Case 2, skin. Individual neoplastic cells are round to oval, with scant cytoplasm and large oval or round nucleus. (H&E)

Following rapid clinical deterioration and onset of anorexia and vomiting, euthanasia was performed on humane grounds. Post-mortem examination, together with cytology and histology, confirmed CTVT in the penile glans, skin of the right neck (Figures 6a & 6b), and metastasis to the liver and mandibular lymph nodes. Retained testicles were absent. This diagnosis was supported by immunohistochemistry (positive staining for vimentin, negative staining for CD3 (T-lymphocyte), CD79a (B-lymphocyte), CD18 (leucocyte)and Iba1 (histiocytic disease)) and *LINE-MYC* PCR amplification.

## Discussion

The increasing popularity of rehoming “rescue dogs” from Eastern Europe has been linked to increasing numbers of CTVT cases in the UK (Gibson *et al*. 2021). Many rescue dogs appear to be incorrectly imported under a companion animal travel scheme which does not require pre-travel health checks (Jarvis 2023; Loeb & Mack 2023; Norman *et al*. 2020). Once in the UK, CTVT may transmit if affected dogs are not isolated and neutered. The location of CTVT in case 2 at the base of the penis indicates that transmission almost certainly occurred during vaginal penetration, despite this animal’s neutered status. We recommend that owners are informed that canine sexual activity and risk of CTVT transmission is not eliminated after castration.

Diagnosis is usually by history, clinical and cytological appearance of the tumour, but when there is uncertainty histology and CD3, CD79a and CD18 immunohistochemistry can be performed (Fernandez *et al*. 2005). In case 2, the diagnosis was assisted by PCR to detect the LINE-MYC rearrangement (Castro *et al*. 2017; Murgia *et al*. 2006). This highly sensitive method can be useful to confirm CTVT using minimal input material. In this case, we were able to use PCR to confirm CTVT using cytology samples obtained from the base of a cavitating neck mass, close to the jugular vein and common carotid artery, and ultrasound guided needle aspirates from the liver. Obtaining samples for histology from both areas would have added procedural morbidity. Archival formalin fixed paraffin embedded material from case 1 were also diagnostically confirmed using LINE-MYC PCR.

It is noteworthy that both cases showed metastasis and chemotherapy resistance. Chemotherapy resistance may be conferred by alteration in tumour genetic or epigenetic state or by failure of the host immune system to execute chemotherapy-triggered immune-mediated tumour regression (Gonzalez *et al*. 2000). Experimental work demonstrated that CTVT is more aggressive in immunosuppressed dogs (Cohen 1973). Although we cannot exclude it, there is no reason to believe that these cases were immune deficient; the dogs were unrelated, lived in a domestic setting, and had good access to veterinary care in comparison to free-ranging street dogs. Thus, we consider it likely that failure of treatment was due to tumour-intrinsic properties. It is possible that tumour-intrinsic chemotherapy resistance and metastatic potential was acquired after initial, partial treatment of case 1, and subsequently passed to case 2. Although there are some preliminary studies on the topic (Alzate *et al*. 2019), there is little understanding of the mechanisms that confer intrinsic chemotherapy-resistance in CTVT.

## Conclusion

Although CTVT is unlikely to become endemic in the UK due to dog control laws and the popularity of neutering, imported dogs pose a risk of disease transmission to UK resident dogs where persistent close proximity is permitted. Metastatic rates in CTVT are low, but treatment is not always successful or straightforward. When presented with a newly imported dog, thorough examination of the external genitalia is advised, including endoscopic examination of the vagina in bitches with haematuria.
